# An unusual clinical variant of systemic lupus erythematosus

**DOI:** 10.11604/pamj.2025.52.86.48125

**Published:** 2025-10-29

**Authors:** Switi Jawade, Savita Pohekar

**Affiliations:** 1 Department of Obstetrics and Gynecology Nursing, Shalinitai Meghe College of Nursing, Datta Meghe Institute of Higher Education and Research (Deemed to be University), Sawangi, Wardha, Maharashtra, India; 2 Department of Medical Surgical Nursing, Shalinitai Meghe College of Nursing, Datta Meghe Institute of Higher Education and Research (Deemed to be University), Sawangi, Wardha, Maharashtra, India

**Keywords:** Systemic lupus erythematosus, multiorgan involvement, autoimmune disease

## Image in medicine

Systemic lupus erythematosus (SLE) is a complex and systemic autoimmune disease in which a person's own immune system attacks their healthy cells and tissue with multi-organ and central nervous system involvement, such as brain, skin, bones, kidney, joints, lungs and blood vessels. The disease is more common in childbearing age in females; it has been well reported in paediatrics and among the elderly. A 24-year-old female visited the dermatology outpatient department with complaints of a fluid-filled lesion (red colour) over the hand and red lesions on the lower extremities with pedal oedema, burning sensation near the lesion, knee, elbow and wrist joint pain. On physical examination, photosensitive rashes were observed, and laboratory investigation showed the patient had systemic lupus erythematosus. The patient was referred to the dermatology department for further medical management.

**Figure 1:**
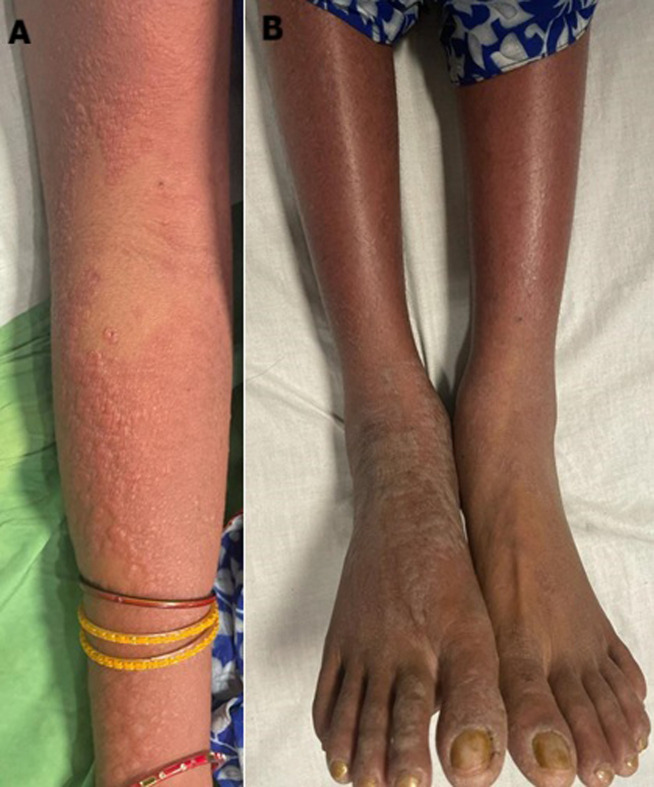
A) erythematous fluid-filled lesion over the hand; B) erythematous lesion over the lower extremities

